# Dr. Mossman's Reply to Mr. Taylor, on Digitalis

**Published:** 1809-04

**Authors:** George Mossman

**Affiliations:** Bradford


### *To the Editors of the Medical and Physical Journal.*

GENTLEMEN,

MR. TAYLOR says, "He does not recollect" that he has reprobated the *digitalis*. I will assume, then, that Mr. Taylor believes that a judicious use of *digitalis* will, sooner or later, controul the action of the heart.

I will further observe, that whatever Mr. Taylor's theory of phthisis may be, he must I think admit, that it is highly desirable to abate the fever of iritation, and thereby send the blood through the lungs with diminished velocity; if so, is there an agent yet discovered, which can with so much certainty, and in the same space of time as the foxglove, effect this purpose? If there be, and Mr. Taylor will poiut it out, I shall be proud to congratulate him, and hail the discovery. In the progress of pulmonary consumption, the principal *wear and tear* of system (if I may be allowed the expression) is occasioned by the increased action of the heart; and therefore it necessarily follows, that what diminishes that action must be more or less subversive of the causes productive of dissolution.

That late very extraordinary young physician, *M. Bichat*, ingeniously defines life to be, "*the union of those functions which resist dissolution.* Living, organized bodies," says he, "are surrounded by agents of destruction. Inorganic bodies act upon them incessantly; and a continual mutual action is set up between them. Were it not for a permanent principle of reaction, death would ensue. This is the principle of life, known only by its phenomena." Now, if the irritative fever be an agent, perpetually operating destructively, another power which can hold this in control, must, necessarily, be preventive to a certain degree of one of the causes of death. The irritative fever is perhaps the cause of all the wasting debilitating tating discharges; and, if so, abating this fever, is accomplishing an object of high import.

Much has been said respecting one action being destructive of another; and much, perhaps, may be achieved by further research.

The action of mercury is confessedly safe, and certainly destructive of some morbid actions. Why should it not then be extended to others? Previous to the introduction of vaccination by the immortal Jenner, I employed mercury in the small-pox; and by its use, I could materially influence the destinies of that disease.

The manufacturing district in which I live, is hardly ever free from typhus fever. For many years I have employed mercury in that disease; and I do not remember one case terminating fatally where mercurial influence was apparent in the mouth. I could adduce an immensity of evidence to establish this fact. A remarkable case lately occurred to me, in which symptoms of the most serious and desperate complexion were not only relieved, but finally cured, by half an ounce of mercurial ointment being daily rubbed into the system. So soon as mercurial action was perceptible in the mouth, did the circumstances of danger begin to disappear. This case, as far as it goes, is in favour of a new action. An ingenious surgeon of this place, a few years ago, attended a young lady in the closing scene of pulmonary consumption. It occurred to him that the lady had never had the small-pox. He vaccinated her. The disease appeared at the usual period, and in the usual form; and it is remarkable, that, for two or three days, the cow-pox seemed to hold sole dominion in the system. The symptoms of phthisis were apparently suspended. The irritative fever was lessened, and the cough disappeared. The cow-pox finished its career, and made way for a recurrence of all the phthisical symptoms. This was certainly the effect of a new action.

If by digitalis the motion of the heart is to be regulated, and if this regulation can be continued for weeks and months, and with safety too, we have reason, a priori, to expect much from its employment.

If *Bichat's* Views of Life and of Death be correct, the objects we are to have in view, are to diminish the efforts of the interior power, and to increase the means of interior resistance.

Having said so much on this subject, I shall now lay before you the authorities of the respectable names I quoted in in my last, in support of the properties and employment of *digitalis.* In the execution of this task, I shall not pursue the path which Mr. Taylor has done. He has turned over a few of the pages of the late Dr. Hamilton; but I entreat my readers only to turn over the first seventy-six pages of that work, and compare them with Mr. Taylor's paper. By this arrangement they will judge if Mr. Taylor has not made partial extracts from this work. Mr. Taylor does not seem to know, that my friend Dr. *Ferriar* ever wrote a Treatise professedly on the subject of *Digitalis*; a work which is well worth Mr. Taylor's perusal; which treats of *digitalis,* and of *digitalis* only.

I have at this moment before me, the books themselves, and all the authorities I have appealed to; and of each of their sentiments, I will present you with a brief but a faithful analysis.

I begin with Dr. *Withering.* He says, that the digitalis will not constantly act as a diuretic; but that it will, more generally, produce this effect, than any other power he is acquainted with. That there is little chance of employing any diuretic after the digitalis has been fruitlessly administered; and that it has often succeeded after every other agent has been tried in vain. That it possesses a power over the heart to a degree unobserved in any other medicine; and that, therefore, it may be employed to atchieve the most salutary ends. That it is so effectual in curing *dropsies,* that it may probably be administered with advantage in particular descriptions of *palsy* and *hydrocephalus,* and in some kinds of *insanity.* That it has been much employed in the West of England, in *phthisis;* and more especially by a very eminent practitioner, Mr. *Saunders,* formerly of Stourbridge. He confesses that, in his own hands, it has not done much in phthisis; but he wishes others to prosecute the inquiry. He concludes, by saying, that, in spite of opinion, prejudice, or error, time will fix the real value upon the discovery, and determine, whether he has imposed upon himself and others, or contributed to the benefit of science and of mankind.

Dr. *Ferriar* says, that he has been careful not to over estimate the power of *digitalis*; that, at present, he regards it as a remedy of the highest class; that its exhibition has become as familiar in his practice, as that of *bark* and *opium;* that it deserves to be ranked with those medicines; that he believes it will be found eminently serviceable in a wide range of diseases; and that an investigation of its effects, promises ample scope for the exercise of of skill and ingenuity; that an extensive employment of it for nine years, has enabled him to speak of its properties with some degree of confidence; that his first trials of it in pulmonary complaints were suggested by the opinions of Dr. *Withering*, Dr. *Darwin*, and Sir *George Baker*; that its effect on the action of the heart was a phenomenon too striking to be overlooked; that it made an impression on his mind, which was strengthened to a conviction of its utility, by a patient and cautious course of observation; that if any man had expressed an opinion a few years ago, that we should discover a medicine capable of reducing the pulse without danger, from one hundred and twenty in a minute to seventy-five or eighty, at the will of the practitioner, he would have been ridiculed as a visionary; that such, however, under proper management, is the power of *digitalis*. That in active *hæmorrhage*, he has always succeeded in reducing the pulse; and generally, in curing the disease. That bleeding is very inadequate to the purpose of lessening the circulation for any considerable time, unless it be carried to a dangerous excess. That the *foxglove* furnishes us with the means of regulating the pulse to our wish, and of supporting a given state of velocity as long as we judge it proper. That after he had established the power of *digitalis* in *hæmorrhage*, he was encouraged to try it in the first stage of *pulmonary consumption*; that it completely removed the symptoms, even during winter, of the very first case in which he employed it; and that he treated several others with at least temporary success. He confesses, however, that in a majority of instances he was disappointed. He adds, that if nothing more was gained by the use of *digitalis* than the mitigation of suffering, we should deem it a valuable part of medical practice in *phthisis pulmonalis*, but that much more benefit may be derived from it. He says, that he believes that *digitalis*, properly administered at the beginning of *phthisical* affections, may suspend the morbid actions of the lungs, by which tubercles are formed; that by its continued exhibition after *hæmoptysis*, it may be possible to procure the cicatrization of the ruptured vessels, and thus to prevent the formation of ulcers. That he is disposed to hope, that its power of soothing irritation may extend so far, as sometimes to heal ulcerations of the lungs in the advanced stage of *consumptions*. That a remedy, from which these expectations may be indulged, is of unspeakable value, and merits the strictest attention of the physician; that in coughs of long standing, the use of the *digitalis* has given more relief than any other medicine which which he has employed. That from the sedative power of *digitalis,* it may be expected to prove highly useful in many cases of *active inflammation,* particularly in *pleurisy* and *peripneumony;* that we have long wanted a remedy capable of lowering the pulse without increasing evacuation to a dangerous degree; that in every species of *Hydrocephalus, digitalis* appears to be indicated; that in *gangrenous* cases, arising from excessive irritability, *digitalis* may be properly combined with *opium;* that in *pulmonary consumption,* arising from *hæmoptysis* or *tubercles,* much relief may be obtained from the use of *digitalis;* and that even a cure may now be hoped for, under circumstances which formerly precluded all expectation of recovery.

So much for *Dr. Ferriar;* and so much for Dr. Mossman's "absurdity" in quoting this excellent and experienced physician.

If Mr. Taylor will purchase and study this interesting little work, he will be amply repaid for his labour; and he will be much delighted by a note from the brilliant pen of the late learned aud philanthropic *Percival.*

Dr. *Beddoes.*—It would far exceed the limits I have prescribed to this paper, were I to quote extensively from my ingenious friend.—Speaking of the agency of *digitalis,* he says, that in cases of *pulmonary* disease, where the presence of tubercles was indicated by every symptom, and where they seemed to break out into open ulcers, he has verified the efficacy of *digitalis;* that he daily sees patients advancing towards recovery, with so firm a pace, that he hopes, that consumption will, henceforward, be as regularly cured by the *foxglove* as *ague* is by the *Peruvian bark;* that could we find an auxiliary for the *foxglove,* that not one case in five would terminate, as ninety-nine in an hundred have hitherto terminated; that a majority of cases will yield to simple *foxglove;* that where he had all possible evidence of the existence of tubercles, the exhibition of the *digitalis* had been perfectly successful; that he under-rates the proportion of favourable events, when he says, that it succeeded in three cases out of five; and that he adheres to the opinions he expressed in his *West Country Contributions,* that robustness of constitution is peculiarly favourable to the action of *digitalis.*

If Mr. Taylor will procure this work, and carefully peruse the cases by Dr. *Magennis,* I think he will be convinced of the value of *digitalis.*

Dr. *Drake* is the next authority I quoted.—He says, that that the experience he has had, has thoroughly convinced him, that the *digitalis* will be found adequate to the cure of several cases of *tubercular consumption,* though advanced into the second stage; that it will, for a time, suspend the progress of the symptoms in almost every instance; and that in *catarrh, hæmoptoe, and vomica,* it will, in some measure, approach to what is commonly implied by the term specific. That the success which has attended its exhibition in *phthisis,* has been considerable; that several in its confirmed state have been cured by this remedy; that almost all have been relieved; that life has been protracted by it; and that when death did take place, whilst the system was under its influence, it has been free from pain and struggle. That he who hastens to employ it early in *tubercular consumption,* will in proportion to his promptitude be a benefactor to mankind.—Vide Med. Journal, vol. ii. p. 118.

Dr. *Maclean* says, that his success in the exhibition of the *Digitalis,* for upwards of five years, has exceeded his most sanguine expectations; that he has found it a most valuable *antiphthisical* remedy; and he trusts, that it will be found by others, the most valuable that has hitherto been resorted to. That its success is in proportion to its early exhibition; that he has been fortunate enough, by means of this remedy, in rescuing a few from death, after every other means had failed; that if it do not cure, it will more or less soothe the sufferings of the patient, lengthen the duration of life, and smooth the avenues to death.—Vide Med. Journal.

Dr. *Thomas,* having quoted Dr. *Fowler,* Dr. *Drake,* Dr. *Beddoes,* and myself, as its chief advocates, says, that from the observations of all these gentlemen, as well as from those of other physicians, and from his own, the *digitalis* must certainly be allowed to be a very powerful remedy in *phthisis;* and, although it is by no means to be regarded as a specific, yet, that it must be admitted, that in many instances it has procured the most beneficial effects, even at a very advanced stage of the disease; that let its power depend upon what they may, certain it is, that its success is proportioned to its early exhibition; and that in every clearly marked *phthisical* case, it ought, without loss of time, to be had recourse to.

The last authority I quoted was the late Dr. *William Hamilton.*

I believe this book is in the possession of Mr. Taylor; and when I have made from it a few questions, Mr. Taylor lor may perhaps, that he may judge of my accuracy, be induced to read it to an end. Dr. *Hamilton* begins with this remarkable passage from *Currie's* Reports.—"This medicine (*Digitalis*) may almost be said to be possessed of a charm for allaying inordinate action of the heart and arteries; and in this point of view, as well as for its efficacy in some kinds of *dropsy*, particularly *hydrothorax*, its introduction into medicine is one of the greatest benefits our science has received in modern times." Dr. *Hamilton* says, that in its progress to the important action, among the means of curing diseases, which it is intitled to occupy, it has serious obstacles to encounter; that its injudicious administration makes it an object of terror to some, and to others an object of indifference; but, that the few who carefully observe its administration and effects, are delighted with the powers which it is often capable of exerting. He gives us a table of fifteen patients treated by Dr. *Drake* with the *digitalis*; by which it appears, that nine were cured by the plant; one was undetermined; and five perished.

He says, that when water is present in some of the cavities of the thorax, we can look only to *digitalis* for decided relief; and should we fail of ultimate success, we are employing the only remedy, which by its singular dominion over the heart, can allay inordinate action, and thus render the feelings of the patient more tolerable. He says, that it is during the early progress of consumption, that we are to expect lasting advantages from the *digitalis*, though he admits, that in the advanced stages it will often relieve symptoms and protract life. That he is of opinion, that few instances of consumption would be found to resist the powers of the *digitalis*, or advance into the second stage, were it resorted to at the very first appearance of symptoms.

I would advise Mr. Taylor to peruse with attention Dr. *Hamilton's* cases in the Appendix; and case the fifth he will find a very remarkable one. It was a very desperate case of *hydrothorax* cured by the *foxglove*; and Dr. *Hamilton* observes, that he was aided in consultation by an intelligent surgeon at *Harleston*, and that Mr. *Strowger* was the man; to which case is subjoined a well-written letter by this Gentleman to Dr. *Hamilton*. Mr. Taylor's examination of Dr. *Hamilton's* work, seems to extend only to the first 76 pages.

Dr. *Hamilton* has a note from Dr. *Maclean*, detailing a very remarkable case, where every other means had fail-

 ed, ed, and where the patient appeared approaching fast to the end of all his sufferings; the *foxglove* was resorted to, and the *foxglove* saved the patient's life. Dr. *Hamilton* finally closes his work by a quotation from Dr. *Maclean*, in which that gentleman says, that he "confidently looks forward to the period, when this valuable medicine will completely triumph over the obstacles, which prejudice and ignorance have hitherto opposed to its progress."

Mr. Taylor has told us, that a great number of *consumptive* patients are met with in his district.—He does not say by whom.—He tells us that the *digitalis* has been usually given.—He does not say by whom,—and he leaves us quite in the dark respecting its effects. I shall now take my leave of Mr. Taylor by assuring him, that I am in perfect good humour; and when he replies to this paper, I hope to find him equally pleasant.

I am, &c.

GEORGE MOSSMAN.

*Bradford, Dec.* 13, 1808.

